# Re-Training of Convolutional Neural Networks for Glottis Segmentation in Endoscopic High-Speed Videos

**DOI:** 10.3390/app12199791

**Published:** 2022-09-28

**Authors:** Michael Döllinger, Tobias Schraut, Lea A. Henrich, Dinesh Chhetri, Matthias Echternach, Aaron M. Johnson, Melda Kunduk, Youri Maryn, Rita R. Patel, Robin Samlan, Marion Semmler, Anne Schützenberger

**Affiliations:** 1 Division of Phoniatrics and Pediatric Audiology, Department of Otorhino-laryngology Head & Neck Surgery, University Hospital Erlangen, Friedrich-Alexander-University Erlangen-Nürnberg, 91054 Erlangen, Germany; 2 Department of Head and Neck Surgery, David Geffen School of Medicine at the University of California, Los Angeles, Los Angeles, CA 90095, USA; 3 Division of Phoniatrics and Pediatric Audiology, Department of Otorhinolaryngology, Munich University Hospital (LMU), 80331 Munich, Germany; 4 NYU Voice Center, Department of Otolaryngology–Head and Neck Surgery, New York University, Grossman School of Medicine, New York, NY 10001, USA; 5 Department of Communication Sciences and Disorders, Louisiana State University, Baton Rouge, LA 70801, USA; 6 Department of Speech, Language and Hearing Sciences, University of Ghent, 9000 Ghent, Belgium; 7 Department of Speech, Language and Hearing Sciences, Indiana University, Bloomington, IA 47401, USA; 8 Department of Speech, Language, & Hearing Sciences, University of Arizona, Tucson, AZ 85641, USA

**Keywords:** convolutional neural networks, re-training, finetuning, high-speed imaging, glottis, voice, concept shifts, catastrophic forgetting, medical image segmentation

## Abstract

Endoscopic high-speed video (HSV) systems for visualization and assessment of vocal fold dynamics in the larynx are diverse and technically advancing. To consider resulting “concepts shifts” for neural network (NN)-based image processing, re-training of already trained and used NNs is necessary to allow for sufficiently accurate image processing for new recording modalities. We propose and discuss several re-training approaches for convolutional neural networks (CNN) being used for HSV image segmentation. Our baseline CNN was trained on the BAGLS data set (58,750 images). The new BAGLS-RT data set consists of additional 21,050 images from previously unused HSV systems, light sources, and different spatial resolutions. Results showed that increasing data diversity by means of preprocessing already improves the segmentation accuracy (mIoU + 6.35%). Subsequent re-training further increases segmentation performance (mIoU + 2.81%). For re-training, finetuning with dynamic knowledge distillation showed the most promising results. Data variety for training and additional re-training is a helpful tool to boost HSV image segmentation quality. However, when performing re-training, the phenomenon of catastrophic forgetting should be kept in mind, i.e., adaption to new data while forgetting already learned knowledge.

## 1. Introduction

**Figure 1.**
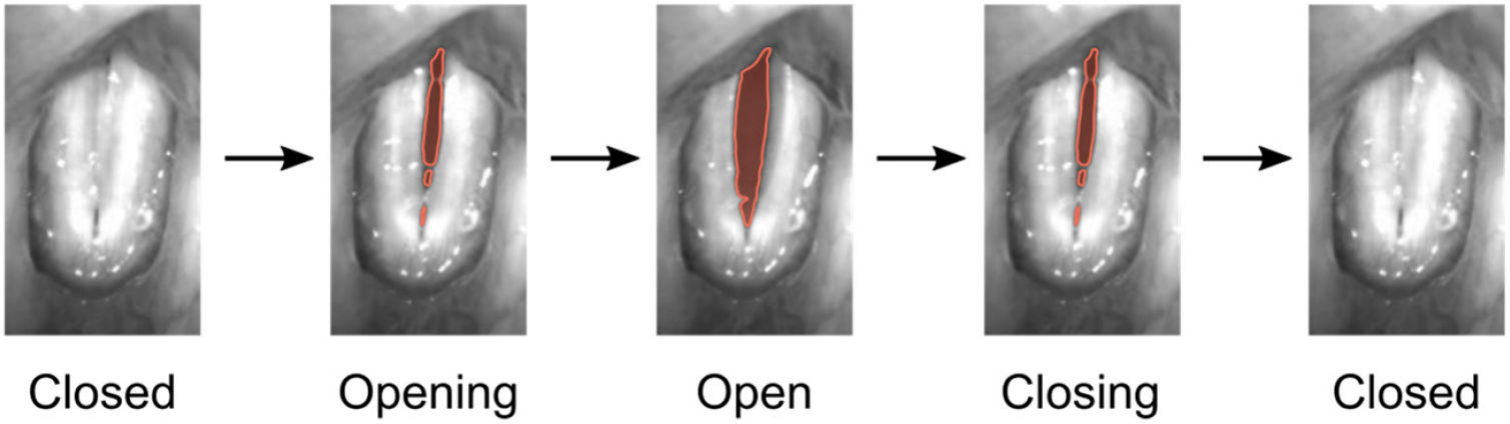
Normal phonatory cycle of the vocal folds recorded by HSV, male subject. The segmented glottis is indicated in red.

Speech and voice disorders become more and more common in the 21st century. The voice is formed by the oscillation of the two vocal folds within the larynx. The vocal folds oscillate on average between 100 Hz (males) and 300 Hz (females) for normal phonation, but can reach up to 1581 Hz during singing [1]. A normal voice or phonation is assumed to be produced by symmetric and periodic vocal fold oscillations [2,3]. Additionally, glottis closure during vocal fold oscillations is assumed to be important for normal voice (see Figure 1) [4]. To capture and assess vocal fold oscillations, digital high-speed video systems have now been used for more than 30 years [5]. Many studies applied HSV imaging to subjectively assess and judge vocal fold vibrations [6,7]. To quantitatively assess and judge vocal fold oscillations in HSV data, image-processing techniques have been suggested to segment the glottal area or detect the vocal fold edges over time (see Figure 1), being requisite for subsequent computation of quantitative parameters [8-12]. The first image-processing approaches go back to the 1990s, where classical image-processing techniques such as reion growing were suggested [13]. Since then, many other classical image-processing techniques as thresholding [14], edge detection [15,16], or active contours [17,13] have been successfully applied. These classical image-processing techniques have been further developed [19] and combined with machine learning methods, e.g., active contours with k-means-clustering [20]. Machine learning methods and especially computationally expensive deep neural networks (DNN) have become more and more popular due to the computational performance increase of computers and, in particular, the effective use of graphics processing units (GPUs) [21]. Specifically, convolutional neural networks (CNNs) based on the U-Net architecture [22] are a popular and commonly used method for glottis segmentation in HSV videos [23-25].

The main advantage of DNNs is that, although they have high computational costs during the training process, they are much faster during application when performing segmentation tasks. Kist et al. [26] reported a <1 min segmentation time for 1000 HSV frames (<0.06 sec/image) for their DNN on a GPU (GeForce GTX 1080 Ti) in contrast to ref. [27], who reported a mean segmentation time of 3.8 sec/image for their fully automated wavelets and active contour-based method on a CPU (Intel^®^ Core ^™^ i5-2400, 2 GB RAM). Although user friendly semi-automatic glottis segmentation is highly reliable [28], the expenditure of time is also significantly higher (approx. 0.9 sec/image) than for current DNNs [26]. Another big advantage is that DNNs are highly reliable even for image quality degradation caused by factors such as blurring or poor light conditions [26]. The current DNN-based methods report segmetation accuracies of over 80%, e.g., refs. [18,24,25]. The current approaches also successfully apply DNNs for automatic glottis midline detection in HSV videos [29]. A comprehensive overview of recent machine learning and DNN approaches fot HSV image segmentation is provided in ref. [21].

**Figure 2.**
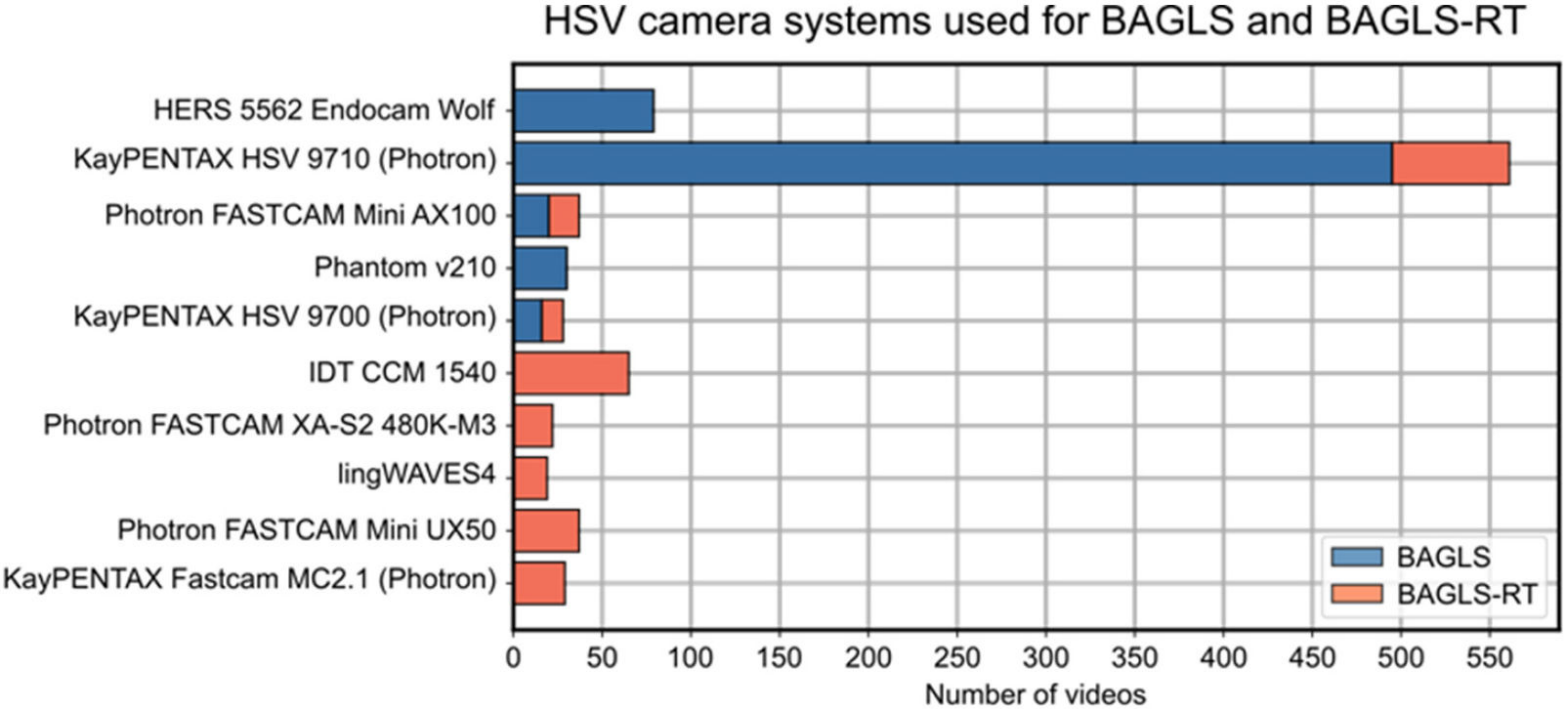
Overview of the considered camera systems and the number of corresponding videos in both data sets. Five new cameras are considered in the BAGLS-RT data set.

To the best of our knowledge, except for the BAGLS data set [30], all previous studies considered only one HSV camera system. Naturally, the trained DNNs may be biased towards other HSV systems using varying camera manufactures (see Figure 2), CCD sensors, spatial resolutions (from 256 × 256 to 1024 × 1024), light sources, and endoscopes. This may be a disadvantage for other researchers or clinicians who want to use existing DNN-based image processing but have different HSV systems than the system the DNN was trained on. In addition, new HSV systems will be developed in the coming years, which will also have different recording modalities, leading to so called “concept drifts” in the resulting images [31]. Especially for these new and hence unknown HSV systems, the segmentation accuracy might significantly decrease, requiring existing DNNs to be adapted [32]. One possibility, although time-consuming, is the (re-)training of a model from scratch [31,33]. The other option is provided by so-called re-training or fine-tuning methods, allowing for easy and fast adaption of existing and pre-trained neural networks.

In this work, we suggested, discussed, and analyzed re-training approaches for HSV image segmentation. To the best of our knowledge, the effect and usefulness of re-training strategies on laryngeal HSV segmentation have not been investigated yet. However, re-training has to be kept in mind and will have to be considered in HSV image processing to enable sufficient accurate segmentation for new camera systems in the future.

## 2. Materials and Methods

### 2.1. Data Set

The BAGLS data set contains 59,250 annotated images from 640 HSV videos. Seven international cooperation partners contributed to the data set, yielding a high diversity in recording modalities. A detailed description of the BAGLS data set can be found in ref. [30].

The new BAGLS-RT data set contains 267 HSV videos from eight different cameras and institutions, yielding 21,050 annotated images. The BAGLS-RT data set expands the BAGLS data set with five new cameras (Figure 2), four new light sources, one flexible endoscope, one new frame rate, and 14 new spatial resolutions, see Tables A1-A5 for details. The subject distribution is as follows: mean age 42 ± 20 years, age range 18–93 years, 177 females and 90 males, 154 patients with healthy voices, and 123 patients with various pathologies, see Table A6. All recordings were performed during sustained phonation.

The BAGLS-RT data set is available at Zenodo (https://doi.org/10.5281/zenodo.7113473 and the BAGLS data set is available at (https://doi.org/10.5281/zenodo.3762320.

### 2.2. U-Net Architecture

#### U-Net (3.2):

**Figure 3.**
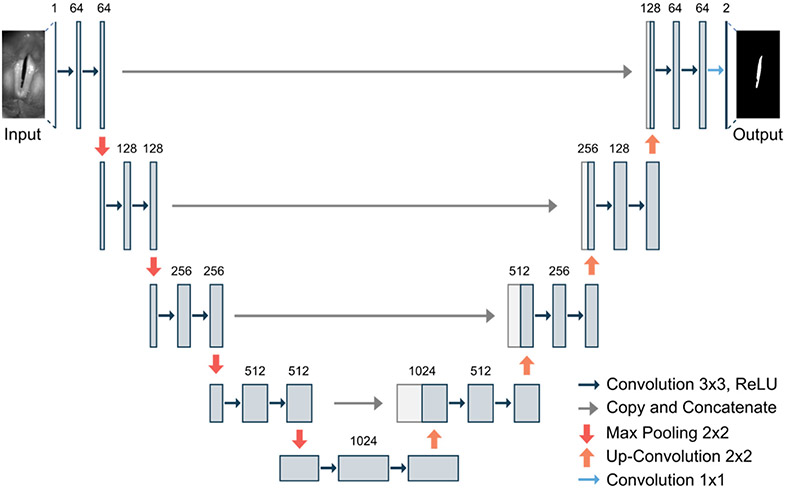
Illustration of the U-Net architecture based on ref. [22].

The U-Net is a commonly used convolutional neural network for image segmentation [22]. Using skip-connections within the encoder–decoder architecture allows for effective and fast learning based on a relatively small data base [34]. The basic structure of the U-Net is illustrated in Figure 3.

In the following, for better understanding for those readers who are not familiar with deep learning, some essential terms are shortly described:

#### Training data:

The data used for training a model on the task, herein glottis segmentation: BAGLS (54,750 images) and BAGLS-RT (18,250 images).

#### Validation data:

During training, the segmentation quality is judged on certain data not being used for training or testing, herein 5% of each training set.

#### Test data:

After the training is finished, the performance evaluation of the final model is performed on so-far unknown test data: BAGLS (4000 images) and BAGLS-RT (2800 images).

#### Batch:

The share of training data that is used for training a model. Batches can contain the entire available training data or parts of it. In this work, we used batch sizes of *b = {25%, 50%, 75%, 100%}* of the available training data within the corresponding BAGLS or BAGLS-RT data.

#### Epoch:

One learning cycle, i.e., adaption or optimization of the U-Net parameters (i.e., parameter update within the U-Net) overall included training data (i.e., the defined batch size). This network parameter optimization (backpropagation algorithm) does not use the entire batch at once, but splits it up in smaller subsets, herein 8 images.

#### Evaluation of segmentation performance:

**Figure 4.**
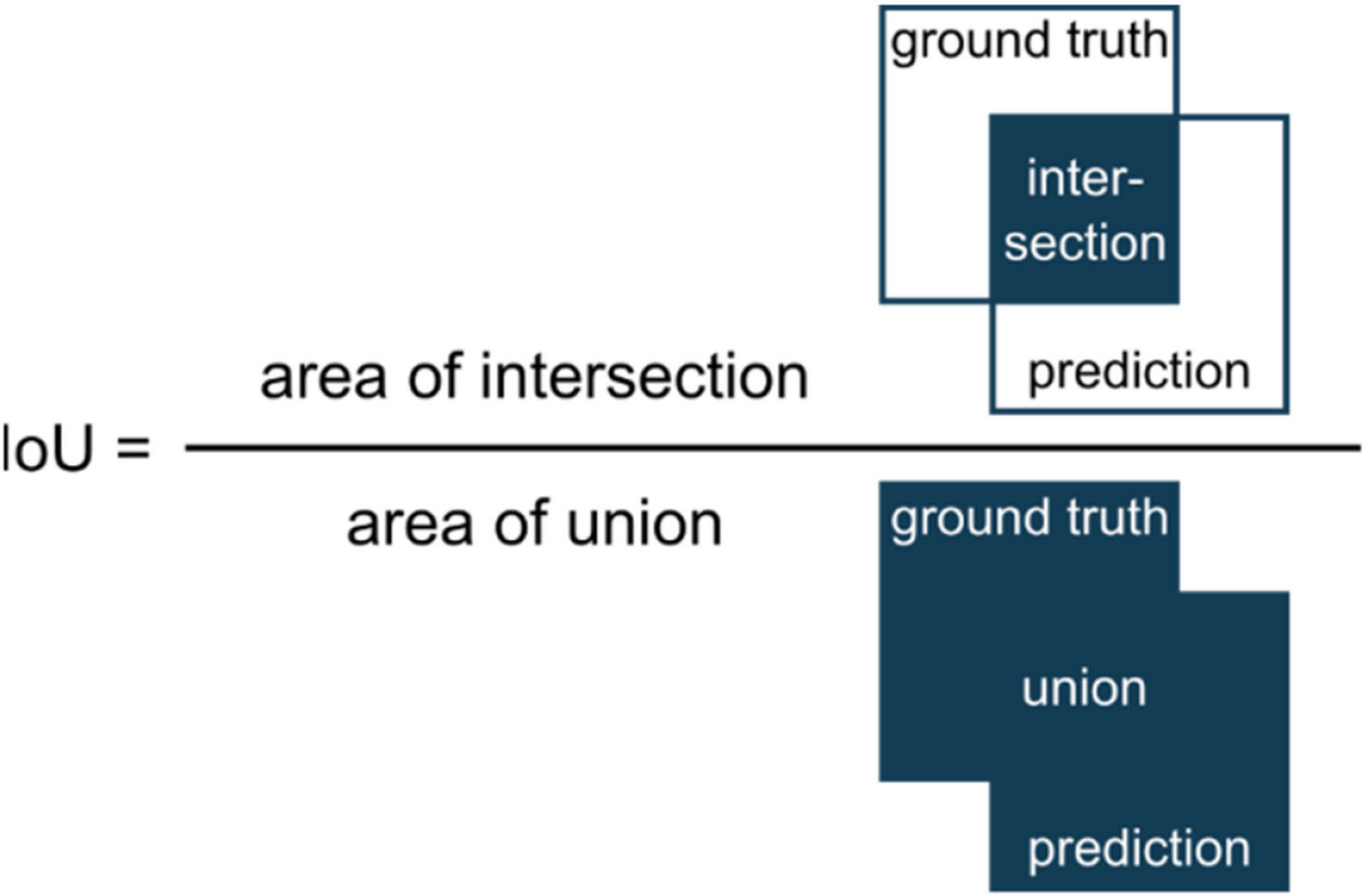
Illustration of the computation of the segmentation metric IoU (Intersection over Union).

For judging image segmentation performance we used the commonly applied Intersection over Union (IoU) [26]. The IoU is a metric that quantifies the overlap between the ground truth (manually annotated data) and the prediction of the U-Net. It divides the overlapping pixels of prediction and ground truth by the sum of all pixels (Figure 4). Thereby, IoU = 1 means perfect prediction.

The U-Net was implemented in Python using TensorFlow 2.5.0 and trained on a NVIDIA GeForce RTX 3080 GPU.

### 2.3. Data Preprocessing

Before training, the images were preprocessed with the following two methods.

#### The U-Net within the TensorFlow framework requires standardized image sizes:

**Figure 5.**
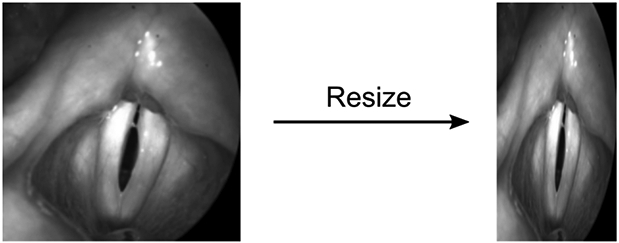
Potential induced deformations using the previously suggested resizing method by ref. [30].

To meet the internal pooling operations of the U-Net, Gomez et al. [30] resized the training and validation images to 512 × 256 pixels (Resize Method). This 2:1 proportion was chosen because it approximates the glottis dimensions. However, this often yielded an undesired deformation of the images resulting in unnatural glottis geometry (Figure 5). Hence, we now suggest a different method called the Region of Interest (ROI) method.

#### Region of Interest Method (ROI):

**Figure 6.**
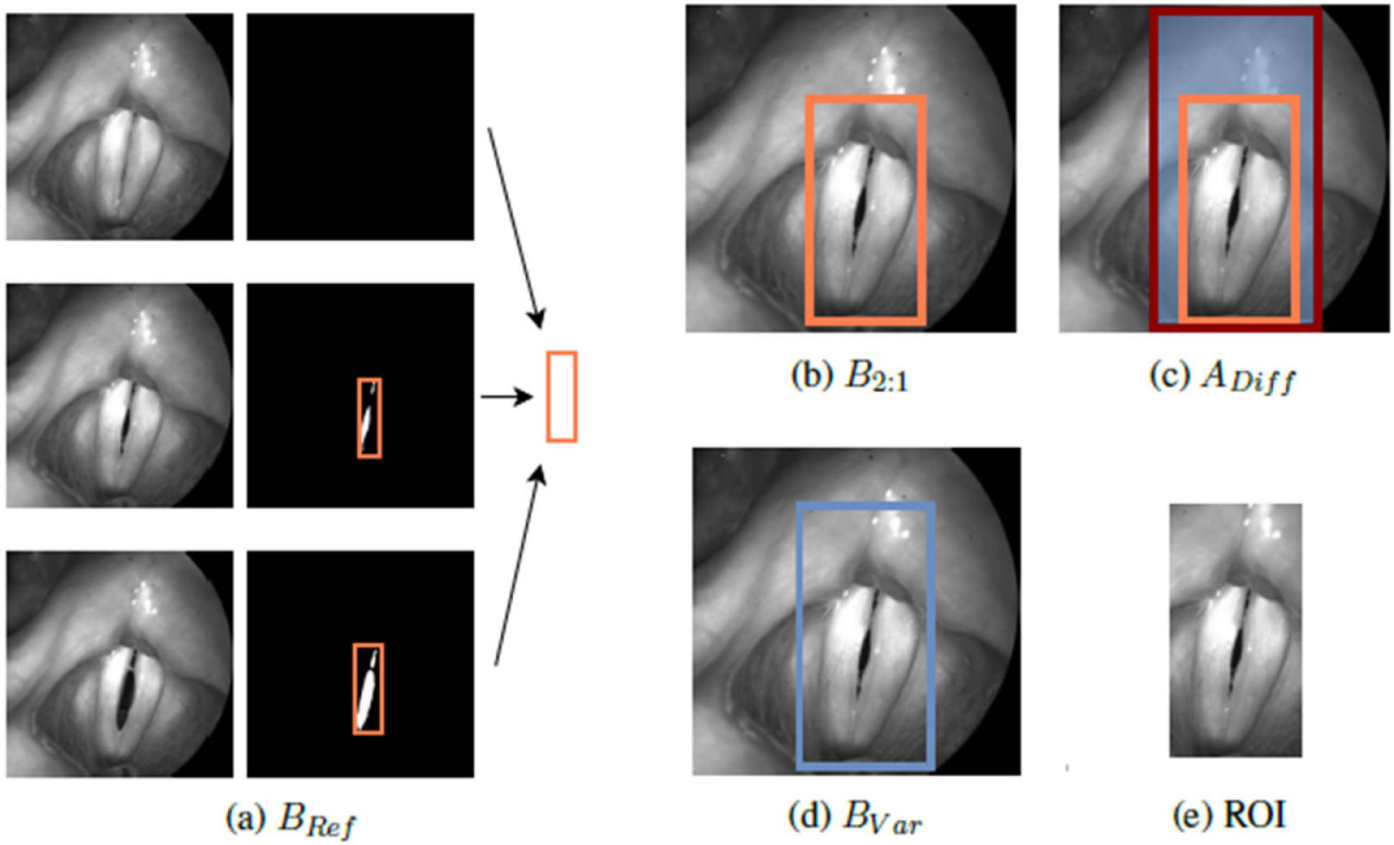
Process of the suggested ROI method in preprocessing for the training data. A randomly chosen ROI is presented in (**e**).

For resizing the images to the desired 2:1 scale (based on the glottis geometry), the following new approach was performed. Within each video, bounding boxes were generated and combined to B_Ref_, enclosing all included segmentation masks (Figure 6a). Afterwards, the smallest (B_2:1_ ≥ B_Ref_) and largest possible bounding boxes in the desired 2:1 scale were determined, defining the boundaries of available ROIs (Figure 6b,c). The region of interest (ROI) for each image may now be an automatically and randomly chosen box B_Var_ within the defined area, yielding more variety in training data regarding the position of the glottis in the image as well as surrounding information (Figure 6d,e).

### 2.4. U-Net Training

#### U-Net training:

If not otherwise specified, hyperparameters were chosen as provided in ref. [30]. First, model parameters were initialized randomly, forming the initial model M_0_. Validation data comprised 5% of training data. A 3-fold cross validation was performed. For model training, an ADAM optimizer with a cyclic learning rate between 10^−3^ and 10^−6^ was used. The mini-batch size was set to 8 images. Training was restricted to max. 100 epochs with early stopping, i.e., if the *Dice Loss* (i.e., overlap of prediction and ground truth) [35] did not improve after 10 epochs on the validation data, training was terminated. Final segmentation quality was then computed over the mean IoU (mIoU) on the test data.

#### Augmentation:

To enhance the variability of the data, images were augmented using Python Package *Albumnetations*. Variations were stochastically performed with brightness and contrast (*p* = 0.75), gamma value (*p* = 0.75), Gaussian noise value (*p* = 0.5), blurring (*p* = 0.5), random rotation between 0° and 30° (*p* = 0.75), and horizontal mirroring (*p* = 0.5) [36]. Augmentation was performed for each epoch during training, yielding different training data for each epoch. In addition, for the previously described ROI method, different ROIs were generated for each epoch. Such augmentation approaches help to avoid overfitting of the model on the training data [37] and may also improve model performance [38].

### 2.5. Re-Training the U-Net

The following re-training strategies were tested to investigate segmentation quality on existing data (BAGLS) and new data (BAGLS-RT).

#### 2.5.1. Re-Training from Scratch

**Figure 7.**
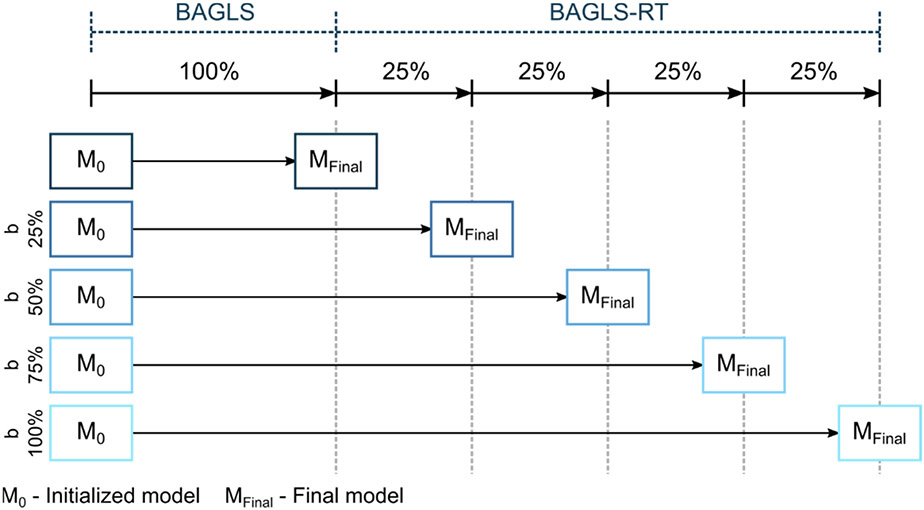
Illustration of re-training from scratch for the different batch sizes.

Here, the entire U-Net was newly trained. For training, both data sets BAGLS and BAGLS-RT were used. The validation data contained 5% of each data set. The BAGLS-RT training set was split in batches *b* of different sizes *b = {25%,50%,75%,100%}* and individually added to the entire BAGLS training set. This allows investigation of how the amount of new BAGLS-RT data influences the training and hence the segmentation performance on BAGLS and BAGLS-RT. The training process is illustrated in Figure 7.

#### 2.5.2. Incremental Finetuning

**Figure 8.**
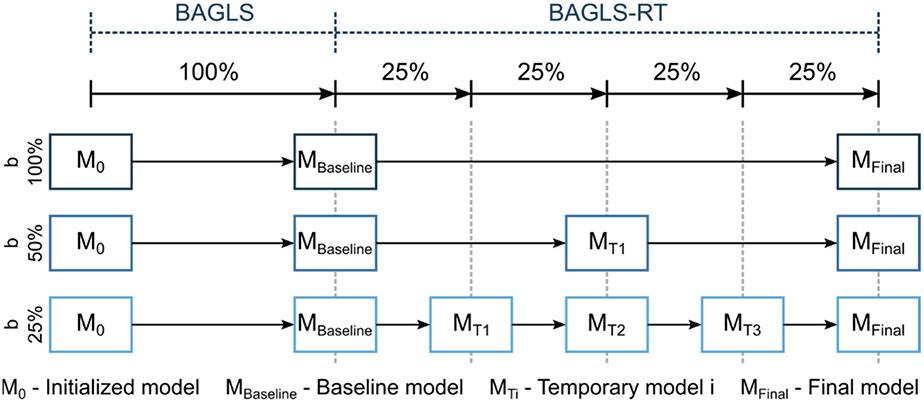
Illustration of the incremental finetuning process for the different batch sizes.

Here, a baseline model, trained solely with BAGLS data, was used as the starting model. Then, only the BAGLS-RT data were used to re-train this model, commonly known as finetuning. To simulate continuous new data, based on this finetuning concept, incremental learning was simulated using different batch sizes *b = {25%, 50%, 100%}* of BAGLS-RT (Figure 8). This means for, e.g., b = 25% that four incremental finetuning steps were performed, as shown in the bottom row of Figure 8.

#### 2.5.3. Incremental Finetuning Using a Mixed Data Set

Again, the baseline model was used as the starting model. Then, the BAGLS and BAGLS-RT data were used to re-train this model using different batch sizes *b = {25%, 50%, 100%}*, where half of the data was from BAGLS-RT and the other half was a representative share of BAGLS data, i.e., for *b* = 100%, the entire BAGLS-RT training set (18,250 images) and 18,250 images from BAGLS were included. This approach uses the same finetuning process as shown in Figure 8 except that the temporary models M_Ti_ are trained with BAGLS and BAGLS-RT. This approach was chosen since considering only new data (BAGLS-RT) in the re-training might yield decreased segmentation accuracy for old data (BAGLS), as will be seen in the Results section for the incremental finetuning abovementioned.

#### 2.5.4. Finetuning with Knowledge Distillation-FKD

**Figure 9.**
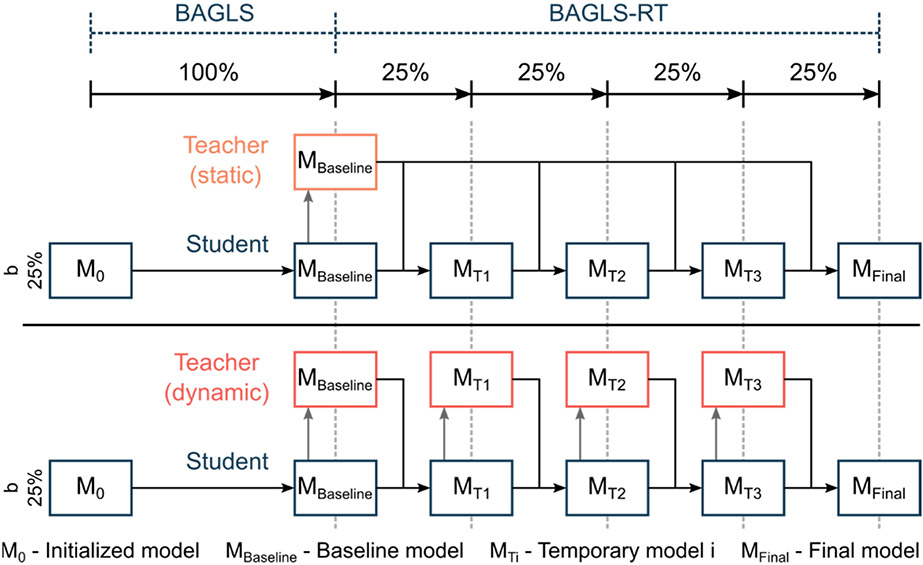
Illustration of the finetuning process using knowledge distillation with a static and dynamic teacher model, exemplarily given for *b* = 25%.

Here, the model performance is judged by considering the *Dice Loss* of both the baseline model previous to re-training (*teacher-model*) and the *Dice Loss* for the model currently being re-trained (*student-model*). The influence of the *teacher-model* is controlled by a parameter α ∈ [0, 1]. The higher the α value is chosen, the smaller the influence of the *teacher-model* during re-training, i.e., α = 1.0 corresponds to the same incremental finetuning as described above in Section 2.5.2. For a detailed description of the FKD approach, we refer to refs. [39-41]. The training data were chosen as described in Section 2.5.2, i.e., only BAGLS-RT. We chose a balanced value with α = 0.5 and investigated three batch sizes *b = {25%, 50%, 100%}*. Additionally, we considered (1) a static model, where the *teacher-model* is always the baseline model, and (2) a dynamic model, where the *teacher-model* is replaced after each batch with the *student-model* (Figure 9). Consequently, the static and dynamic model for the batch size *b* = 100% are equivalent.

## 3. Results

### 3.1. Resizing of the Images

In order to compare the different resizing methods, the U-Net was trained from scratch, as described above, using only the BAGLS data set. Using the ROI preprocessing on training data we achieved a mIoU = 0.7737 ± 0.0029 for the original BAGLS test set as well as a mIoU = 0.7675 ± 0.0024 for the BAGLS test set preprocessed with the ROI method. In contrast, preprocessing the training data with the Resize method by ref. [30] yielded a mIoU = 0.7500 ± 0.0065 and mIoU = 0.7040 ± 0.0078 for the original and ROI preprocessed BAGLS test set, respectively. In both instances, the new ROI method was able to improve segmentation performance. The mIoU was increased by 2.37% (original test data) and 6.35% (ROI test data). In the following, both training and test data are preprocessed by the ROI method. We chose the model with the median mIoU on the BAGLS validation data as **baseline model** for all following re-training comparisons: **mIoU** = 0.7642 (BAGLS test data, ROI preprocessing) and mIoU = 0.7354 (BAGLS-RT test data, ROI preprocessing).

### 3.2. Re-Training from Scratch

Results of re-training are provided in Table 1. Whereas the improvement (ΔmIoU) for BAGLS always was between 0.98% and 1.26%, the segmentation performance for BAGLS-RT continuously improved up to 1.66%, showing a correlation between batch size and segmentation quality. These results demonstrate that the accuracy of the U-Net increases for BAGLS-RT with increasing BAGLS-RT training data, while keeping the accuracy for BAGLS at a similarly high value above baseline accuracy. The increase in accuracy for BAGLS may most likely be based on new information from the BAGLS-RT data set that was not considered or neglected during the training of the baseline model.

### 3.3. Incremental Finetuning

Results of re-training are provided in Table 2. The mIoU improved for BAGLS-RT and was highest with the batch size of b = 50%. However, for both other batch sizes the improvement was rather similar. The decrease of mIoU for the BAGLS data is not surprising, since the training data only consisted of BAGLS-RT data and hence the model adapted more to this new data. Although this decrease is rather small, such phenomena are called catastrophic forgetting and should be avoided in re-training. This phenomenon also illustrates existing differences in the images between both data sets, as assumed above in Section 3.2. Here, due to the small decrease for BAGLS, it can be stated that the data were not significantly different. However, when considering highly different images for re-training, the catastrophic forgetting may significantly reduce the segmentation quality for the original data by “overwriting” original network parameters during the finetuning process.

### 3.4. Incremental Finetuning Using a Mixed Data Set

Results of re-training are provided in Table 3. The highest improvement for mIoU was achieved for both data sets for the batch size b = 100%. By including BAGLS data in the re-training process, catastrophic forgetting could be avoided. However, there was also a smaller increase of mIoU for BAGLS-RT as compared with the incremental finetuning, indicating that including BAGLS training data also hindered better adaption of the models towards BAGLS-RT data.

### 3.5. Finetuning with Knowledge Distillation (FKD)

Results of re-training with the dynamic and static teacher model are provided in Tables 4 and 5, respectively. For the dynamic model, the best segmentation performance for BAGLS-RT was achieved with batch size b = 25%. For BAGLS, the improvement was always similar being around 0.9%. In contrast, for the static model the best values were achieved with b = 100% (BAGLS-RT) and b = 50% (BAGLS). Training with batch size b = 25% showed the lowest improvement for BAGLS. Overall, the static model achieved slightly higher improvements for BAGLS than the dynamic model, since for the static model the parameters of the teacher model were not updated (i.e., remained as the baseline model) and should hence still be “optimal” for the BAGLS data set. In contrast, the improvements for BAGLS-RT were higher for the dynamic model, since the teacher model is continuously updated with additional information of BAGLS-RT.

In summary, finetuning with dynamic knowledge distillation showed the best results over all re-training methods when considering segmentation performance for BAGLS and BAGLS-RT. The main advantage of this approach is that no old data have to be included in the re-training process in order to still consider old information when adjusting to new data. Additionally, new data may be continuously fed to the existing model (e.g., in several batches), allowing for fast adaption towards new recording modalities.

**Figure 10.**
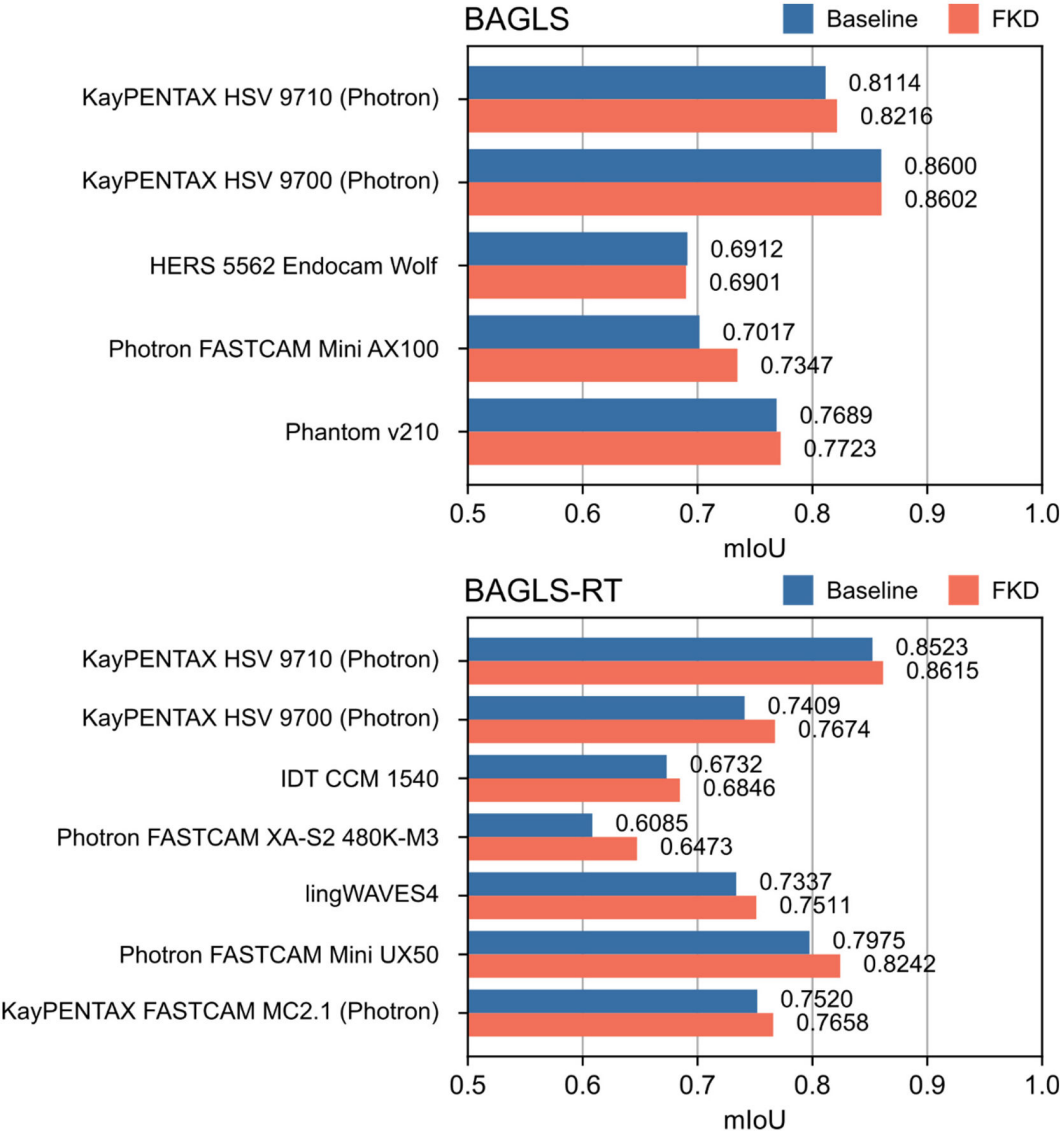
Segmentation performance (IoU) of the baseline U-Net and the U-Net after finetuning with knowledge distillation (b = 100%) for each camera system within the BAGLS and BAGLS-RT datasets.

Since the FKD approach showed the best re-training performance, additional results are provided separately for each camera system. Figure 10 shows the results of finetuning with knowledge distillation with a batch size of b = 100% (i.e., dynamic and static teacher model are equivalent) for each camera system in the BAGLS and BAGLS-RT datasets. Compared with the baseline model, FKD achieved an increase in segmentation performance for all camera systems with the exception of one camera in the BAGLS dataset. Overall, improvements in mIoU were larger for camera systems in the re-training dataset BAGLS-RT (0.92% to 3.88%) than for BAGLS (0.03% to 1.02%). The highest increase in performance (ΔmIoU = 3.88%) could be observed for the Photron FASTCAM XA-S2 480K-M3, which was the only system used in combination with a flexible endoscope, yielding what are arguably the most novel data for the segmentation model, as videos recorded with flexible endoscopes were not included in BAGLS.

## 4. Discussion

Several methods for re-training purposes were discussed and applied to HSV data. In summary, the results showed that diverse training data already enables the model to deal with new modalities to a large amount. Here, this was achieved by random ROI selection and image augmentation. Although smaller than achieved by the new ROI preprocessing method, the subsequent re-training methods showed further improvements. When re-training is performed, the phenomenon of catastrophic forgetting should be kept in mind. Results showed that finetuning with dynamic knowledge distillation seems most promising for re-training with laryngeal HSV data, even outperforming re-training from scratch. Further, this re-training strategy is rather convenient, since no old data are necessary for re-training and therefore do not have to be stored. However, it is also evident that re-training with new data, being not significantly different than the existing training data, can be avoided when the first model training is already based on data with great variety. As Figure 10 shows, re-training for HSV data provided the best results when new data with significant modality changes were considered, e.g., flexible endoscopic HSV data with honeycomb patterns induced by the light fibers.

Regarding future model adjustments, finetuning with knowledge distillation can be adapted depending on the use case. Results showed that using FWD with a static or dynamic teacher model seems to be beneficial towards old or new data due to the respective adjustment procedure for the teacher model. Therefore, depending on whether users prioritize old or new camera systems, a static or dynamic model should be selected. Secondly, the overall influence of the teacher model is controlled by the parameter α. For this paper, we chose a balanced value of α = 0.5. However, if the performance of the model is to be increased primarily for old or new data, this value of α can be decreased or increased accordingly.

The segmentation models resulting from this work will be integrated in the Glottis-Analysis-Tools (GAT) [26] and OpenHSV [11] and made available for other research groups. A limitation of the study is that the used U-Net may have too few parameters (i.e., the model is too simple) to achieve further performance improvements by incorporating new training data. Hence, future work may concentrate on more complex deep learning models containing more parameters [42] that may then lead to further improvement of segmentation performance utilizing additional information in the HSV images that has not been considered by the U-Net. Additionally, deep learning approaches should be applied to the three-dimensional dynamics of the vocal folds [43], potentially enabling an improved insight on the correlation of vocal fold dynamics and acoustic voice quality.

## Figures and Tables

**Table 1.** Segmentation performance of the U-Net after re-training from scratch on the corresponding test data of BAGLS and BAGLS-RT.

Training with Batch Size b	mIoU		ΔmIoU (Baseline)	
	BAGLS	BAGLS-RT	BAGLS	BAGLS-RT
Baseline	0.7642	0.7354	-	-
b = 25%	0.7763 ± 0.0056	0.7449 ± 0.0019	1.21%	0.95%
b = 50%	0.7740 ± 0.0032	0.7504 ± 0.0099	0.98%	1.50%
b = 75%	**0.7768 ± 0.0031**	0.7497 ± 0.0025	**1.26%**	1.43%
b = 100%	0.7767 ± 0.0043	**0.7520 ± 0.0057**	1.25%	**1.66%**

**Table 2.** Segmentation performance of the U-Net after incremental finetuning with BAGLS-RT.

Training with Batch Size b	mIoU		ΔmIoU (Baseline)	
	BAGLS	BAGLS-RT	BAGLS	BAGLS-RT
b = 25%	0.7495 ± 0.0025	0.7597 ± 0.0011	−1.47%	2.43%
b = 50%	0.7514 ± 0.0042	0.7609 ± 0.0029	−1.28%	**2.55%**
b = 100%	0.7563 ± 0.0055	0.7571 ± 0.0029	**−0.71%**	2.17%

**Table 3.** Segmentation performance of the U-Net after incremental finetuning with mixed data from BAGLS and BAGLS-RT.

Training with Batch Size b	mIoU		ΔmIoU (Baseline)	
	BAGLS	BAGLS-RT	BAGLS	BAGLS-RT
b = 25%	0.7694 ± 0.0079	0.7498 ± 0.0020	0.52%	1.44%
b = 50%	0.7644 ± 0.0051	0.7491 ± 0.0016	0.02%	1.37%
b = 100%	**0.7715 ± 0.0035**	**0.7520 ± 0.0011**	**0.73%**	**1.66%**

**Table 4.** Segmentation performance of the U-Net after incremental finetuning with knowledge distillation and the dynamic teacher model.

Training with Batch Size b	mloU		ΔmIoU (Baseline)	
	BAGLS	BAGLS-RT	BAGLS	BAGLS-RT
b = 25%	0.7729 ± 0.0002	**0.7635 ± 0.0024**	0.87%	**2.81%**
b = 50%	0.7726 ± 0.0048	0.7633 ± 0.0033	0.84%	2.79%
b = 100%	**0.7732 ± 0.0019**	0.7566 ± 0.0006	**0.90%**	2.12%

**Table 5.** Segmentation performance of the U-Net after incremental finetuning with knowledge distillation and the static teacher model.

Training with Batch Size b	mIoU		ΔmIoU (Baseline)	
	BAGLS	BAGLS-RT	BAGLS	BAGLS-RT
b = 25%	0.7654 ± 0.0055	0.7526 ± 0.0059	0.12%	1.72%
**b = 50%**	**0.7742 ± 0.0034**	0.7551 ± 0.003	**1.00%**	1.97%
b = 100%	0.7732 ± 0.0019	**0.7566 ± 0.0006**	0.9%	**2.12%**

## Data Availability

The BAGLS-RT data set is available at Zenodo (https://doi.org/10.5281/zenodo.7113473) and the BAGLS data set is available at (https://doi.org/10.5281/zenodo.3762320).

## Appendix A

**Table A1.** Framerates and corresponding number of HSV movies in BAGLS and BAGLS-RT.

Frame Rate (Hz)	BAGLS	BAGLS-RT	# Movies
1.000	21	17	38
2.000	17	12	29
3.000	30	-	30
4.000	542	214	756
5.000	1	-	1
6.000	2	-	2
8.000	26	2	28
10.000	1	-	1
20.000	-	22	22

**Table A2.** Distribution of HSV data by institutions.

Institution	BAGLS		BAGLS-RT	
	Training	Test	Training	Test
Boston University	10	10	17	-
Louisiana State University	15	10	5	7
New York University	14	10	4	-
Sint-Augustinus Hospital, Wilrijk	30	10	21	-
University of California Los Angeles	20	10	-	-
University Hospital Erlangen	448	20	69	15
University Hospital Munich (LMU)	23	10	12	10
Indiana University	-	-	56	10
University of Arizona	-	-	27	14
**# HSV videos**	**560**	**80**	**211**	**56**
**# HSV images**	**54.750**	**4.000**	**18.250**	**2.800**

**Table A3.** Considered light sources.

Light Source	BAGLS	BAGLS-RT	# Movies
300 watts Xenon	79	66	145
CUDA Surgical E300 Xenon	40	21	61
KayPENTAX Model 7152A	-	41	41
KayPENTAX Model 7152B	491	21	512
Olympus CLV-U20	-	12	12
Storz LED 300	-	65	65
lingWAVES4	-	19	19
unknown	30	-	30

**Table A4.** Included spatial resolutions of HSV cameras.

Spatial Resolution (px) (HxB)	BAGLS	BAGLS-RT	# Movies
1024 × 1024	-	65	65
1164 × 512	-	1	1
882 × 512	-	1	1
880 × 512	-	1	1
873 × 1048	-	1	1
878 × 512	-	3	3
868 × 512	-	1	1
862 × 512	-	1	1
608 × 608	-	19	19
512 × 512	2	8	10
512 × 256	431	72	503
512 × 128	22	2	24
512 × 96	1	-	1
420 × 512	-	2	2
416 × 512	-	1	1
352 × 256	11	-	11
352 × 208	30	-	30
334 × 512	-	1	1
320 × 384	-	21	21
320 × 256	33	21	54
288 × 128	7	-	7
256 × 256	88	17	105
256 × 120	15	12	27
240 × 240	-	17	17

**Table A5.** Included endoscope types.

Endoscope Type	BAGLS	BAGLS-RT	# Movies
Oral 70°	543	228	771
Oral 30°	46	-	46
Nasal 2.4 mm	9	6	15
Nasal 3.5 mm	12	11	33
Nasal, flexible	-	22	22
unknown	30	-	30

**Table A6.** Overview of subjects by pathology.

Subject Status	BAGLS	BAGLS-RT	# Movies
Healthy	380	154	534
Muscle tension dysphonia (MTD)	139	18	157
Muscle atrophy	43	18	71
Unknown	50	-	50
Polyp	9	17	26
Edema	14	11	25
Nodules	13	3	16
Paresis	4	12	16
Cyst	6	10	16
Glottis insufficiency	14	1	15
Others	8	4	12
Contact granuloma	5	2	7
Laryngitis	4	1	5
Scar, sulcus	-	5	5
Leukoplakia	1	1	2
Carcinoma	1	-	1
Papilloma	1	-	1
